# Cloud-Based System for Effective Surveillance and Control of COVID-19: Useful Experiences From Hubei, China

**DOI:** 10.2196/18948

**Published:** 2020-04-22

**Authors:** Mengchun Gong, Li Liu, Xin Sun, Yue Yang, Shuang Wang, Hong Zhu

**Affiliations:** 1 Institute of Health Management Southern Medical University Guangzhou China; 2 Nanfang Hospital Southern Medical University Guangzhou China; 3 Chinese Evidence-Based Medicine Center West China Hospital Sichuan University Chengdu China; 4 Institutes for Systems Genetics West China Hospital Sichuan University Chengdu China

**Keywords:** COVID-19, cloud system, syndromic surveillance, clinical decision support, stakeholders involvement, pandemic, medical informatics

## Abstract

**Background:**

Coronavirus disease (COVID-19) has been an unprecedented challenge to the global health care system. Tools that can improve the focus of surveillance efforts and clinical decision support are of paramount importance.

**Objective:**

The aim of this study was to illustrate how new medical informatics technologies may enable effective control of the pandemic through the development and successful 72-hour deployment of the Honghu Hybrid System (HHS) for COVID-19 in the city of Honghu in Hubei, China.

**Methods:**

The HHS was designed for the collection, integration, standardization, and analysis of COVID-19-related data from multiple sources, which includes a case reporting system, diagnostic labs, electronic medical records, and social media on mobile devices.

**Results:**

HHS supports four main features: syndromic surveillance on mobile devices, policy-making decision support, clinical decision support and prioritization of resources, and follow-up of discharged patients. The syndromic surveillance component in HHS covered over 95% of the population of over 900,000 people and provided near real time evidence for the control of epidemic emergencies. The clinical decision support component in HHS was also provided to improve patient care and prioritize the limited medical resources. However, the statistical methods still require further evaluations to confirm clinical effectiveness and appropriateness of disposition assigned in this study, which warrants further investigation.

**Conclusions:**

The facilitating factors and challenges are discussed to provide useful insights to other cities to build suitable solutions based on cloud technologies. The HHS for COVID-19 was shown to be feasible and effective in this real-world field study, and has the potential to be migrated.

## Introduction

The outbreak of the coronavirus disease (COVID-19) in China and many other countries has put huge pressure on the health care system [1]. One method of controlling the communicable diseases is the use of a surveillance system to track the exposed and infected individuals, as well as clinical outcomes [2-6]. However, traditional surveillance systems have limitations in terms of timeliness, spatial resolution, and scalability [7]. Meanwhile, reporting from these systems tends to be national or regional with insufficient information about diseases at the community or city level, which caused low efficiency for the social distancing and quarantine measures [2,8]. This is particularly true for COVID-19 surveillance for the Hubei, China, where many cases and isolated populations have challenged systems of manual reporting and tracking [9-12].

In response to this significant challenge, we developed the Honghu Hybrid System (HHS) as a pilot for COVID-19 surveillance and control, which was successfully deployed within 72 hours in Honghu in the Hubei province, a city 145 kilometers (90 miles) away from Wuhan (the capital city of the Hubei province) with a population of over 900,000 people. The HHS integrated data from both traditional sources such as case report systems and diagnostic labs, as well as nontraditional sources including structured electronic medical records and social media on mobile devices. The real time acquisition and analysis of highly resolved digital data provide detailed information on symptoms, psychological status, contact history, social behavior, and the physical environment [4,6,13].

## Methods

### Environment and Hardware

Cloud-based hardware provided an efficient solution to solve the problems unique to the COVID-19 epidemic and effectively mitigated issues such as shortage of local technical support, unavailability of experts and physical hardware due to blocked transportation, rapidly changing needs related to functionality, and connections with multiple sources across different platforms. See Multimedia Appendix 1 for details of the virtual machine settings.

### Data Collection

**Figure 1:**
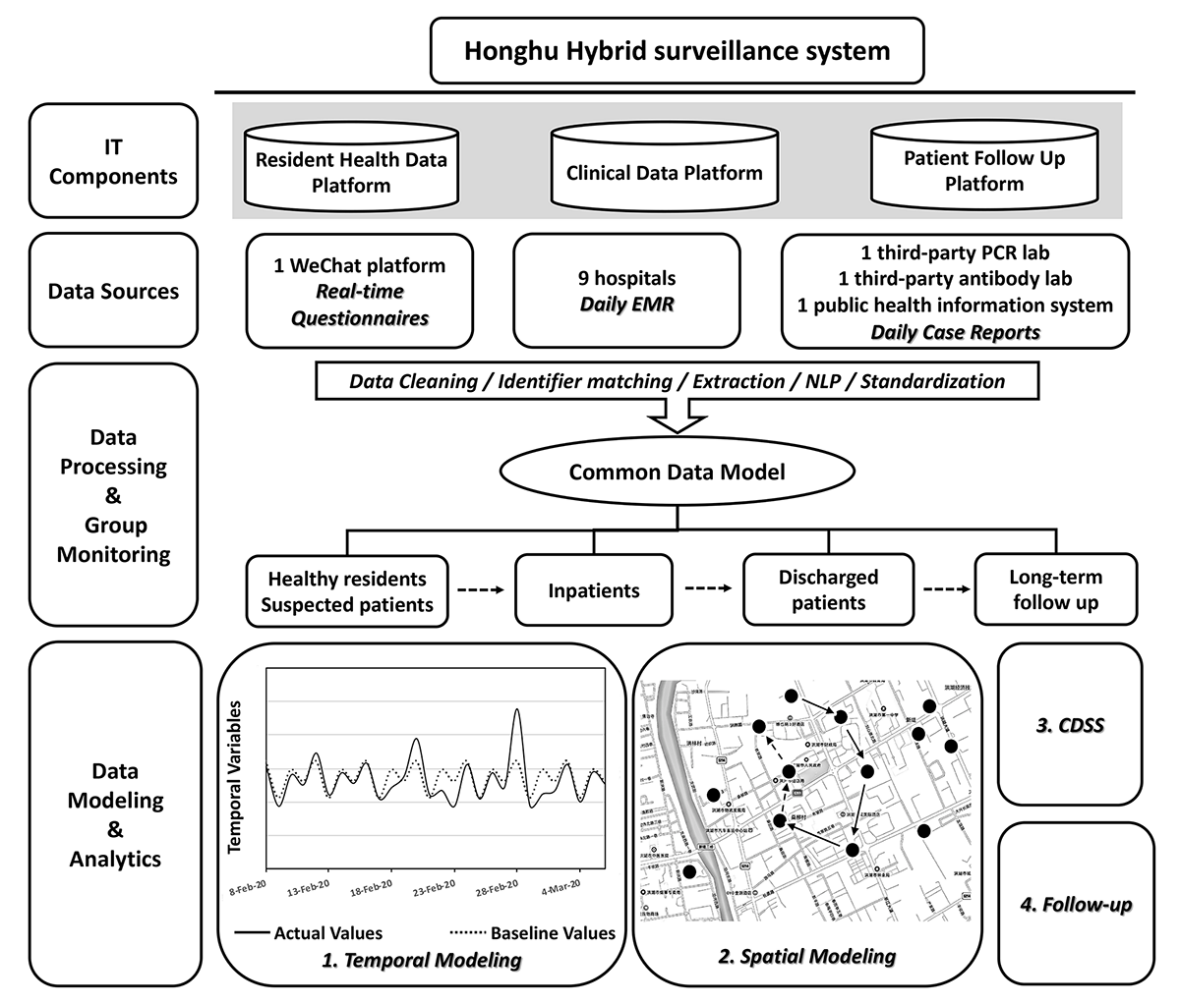
Schematic representation of data streams, processing, and analytics in the Honghu Hybrid System for the coronavirus disease surveillance and control. CDSS: clinical decision support system; EMR: electronic medical records; IT: information technology; NLP: natural language processing; PCR: polymerase chain reaction.

This system collected daily structured electronic medical record data from nine hospitals; real time information about symptoms and personal contact history from the WeChat platform (one of the largest mobile social network apps in China with more than 1 billion monthly active users); and daily reported case diagnosis information from one third-party polymerase chain reaction lab, one third-party antibody lab, and one public health information system (Figure 1). For the data collection, we leveraged existing health information systems inside the nine hospitals and developed a novel mini program for the WeChat platform software development kit for symptom reporting and spatial data collection.

### Data Processing

The data feeds were normalized temporally and spatially and then loaded into a common data model that had been built for the storage, management, and analysis of the integrated COVID-19 data. Vocabulary control was implemented based on the Systematized Nomenclature of Medicine-Clinical Terms synonyms in Chinese for symptoms and the disease itself [14]. Logical Observation Identifiers Names and Codes were adopted to code-related tests. The International Classification of Diseases (ICD) (ie, ICD-10 Clinical Modification) codes were used for the diseases based on the coding standards released by the National Health Commission of China [15,16]. The elements for the data model and recommended synonyms for COVID-19 were collected from 54 Chinese experts, including clinical doctors, researchers, public health professionals, and informaticians.

### Ethics Review, Privacy Protection, and Data Security

The Nanfang Hospital Ethics Committee approved this study. All the users on the syndromic surveillance system consented sharing the necessary information for using this system. Data security and privacy protection were particularly emphasized with administrative as well as technical support [17] throughout the deployment of the system. Standard security settings and software (eg, firewalls, data encryption) were implemented. A virtual private network was used for remote technical support and data analytics. To protect the privacy of patients, we set different levels of access to the data. Aggregated reports were the main form for data sharing. The expert team in the Honghu Municipal COVID-19 Control Headquarter had the highest level of data access. They used the data to triage the patients, coordinate social workers in communities, and allocate the health care resources. The remote analytics team had no access to the identity of the patients. The different levels of access to the data were designed and set up at the beginning of the project by the experts in data security and privacy protection, who have been certified by the Health Insurance Portability and Accountability Act of 1996 security certification. An independent auditor was responsible for monitoring any violation of the rules, as well as reporting and correcting them. Written informed consent was obtained from the inhospital patients before enrollment when data were collected retrospectively. It is important to note that all authorized personnel received training on data security and privacy protection, and additionally, signed legally binding affidavits. In addition, the team worked closely with the cloud computing service provider to separate sensitive data from the rest of the data.

## Results

### Syndromic Surveillance on Mobile Devices

From January 26, when the first case was diagnosed in Honghu, to March 16, 2020, when all the patients with confirmed COVID-19 were discharged, there were in total 383 COVID-19 cases, of which 13 died, unfortunately. Under the huge pressure, HHS was initiated on February 14 and successfully deployed within 72 hours. The accumulated number of self-reports reached 17.5 million by March 16 and the maximum daily active reports reached 900,000 person-times.

Syndromic surveillance was implemented on a mobile phone–based social media platform targeting different groups of individuals. This included the general population, inhospital and discharged patients, people with higher risk of infection (ie, those with travel history to Wuhan, contact history with confirmed cases, or under medical observation in isolation sites), and health care professionals (ie, doctors, nurses, public health experts, and social workers). See Textbox 1 for details of the questionnaire for the general population who was quarantined at home. The items included in the questionnaire had been proposed by infectious disease experts from Nanfang Hospital. Their suggestion was then discussed and modified in a meeting joined by local government staff, local clinical doctors, public health professionals, and informaticians. The items concerning job-seeking support were added to the questionnaire on March 2 when the government was evaluating the release of the restriction on work and production in Honghu. Social workers followed up with over 10,000 positive reports (eg, “I had a temperature of over 37.3 °C” or “I had a severe cough today”) via phone call or home visits. More than 30 individuals were assisted in going to the fever clinic for further screening and then quarantined. This was an active surveillance mechanism initiated by the city residents through new information channels and was effective in COVID-19 screening. The high coverage (over 95% of the residents) and daily active reports (over 600,000 person-times) demonstrated the feasibility of intense monitoring during the COVID-19 epidemic. The stable trends of positive reports (0.10%-0.12% after the daily reports exceeded 500,000 persons) provided the strong evidence that the countermeasures in place were effective in preventing the local outbreaks.

**Real time questionnaire on WeChat.**

**Demographic characteristics**

- Name
- ID number
- Gender
- Address (semi-structured)
- Phone number (free text)

**Spatial information**

- Current location (structured)

**Epidemiological exposure**

- Have you visited or stayed in communities with confirmed cases in the last 14 days? (Yes/No)
- Have you been in contact with confirmed cases in the last 14 days？ (Yes/No)
- Have you been in contact with residents from Wuhan City or from the community with case reports or who have had respiratory symptoms in the last 14 days? (Yes/No)
- Have you participated in small-scale gatherings with more than 2 cases reported in the last 14 days? (Yes/No)

**Physical conditions**

- Today's physical condition: good/cough/runny nose/chest tightness/diarrhea/muscle soreness? (multiple choices)
- Today's body temperature?

**Psychological conditions**

- Did you feel nervous, fearful or anxious today? (Yes/No)

**Community support**

- Are there public health officials to visit you for investigation? (Yes/No)
- Were there any social workers who called you or came to your home to help solve the reported problems? (Yes/No)

**Job-seeking support**

- Are you willing to work locally? (free text)
- Are you willing to participate in free skill training? (free text)

### Policy-Making Decision Support

**Figure 2:**
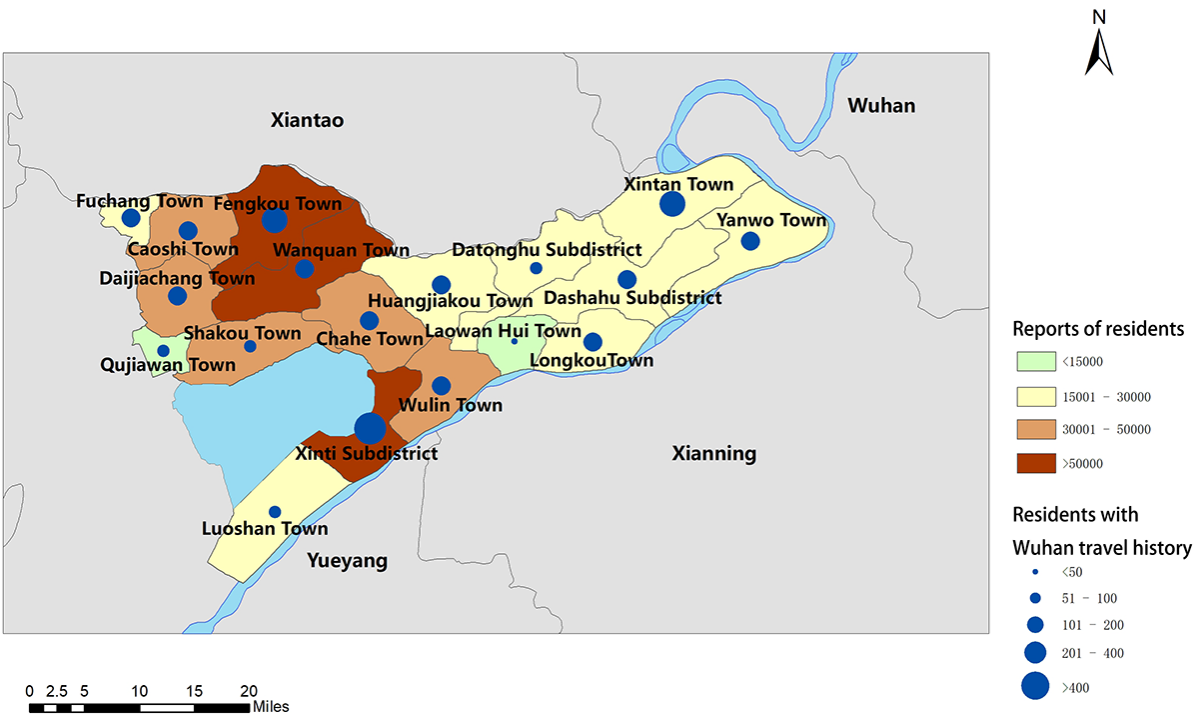
Honghu health reports heat map.

Monitoring the fluctuation and trends analysis of the syndromic surveillance data supported policy-related decision making. Due to the large population size, stability and fluctuation of the trends provided strong evidence for local authorities to evaluate the effectiveness of disease management and make timely adjustments accordingly. Spatial analyses also played a critical role. Clustering of exposed residents inside a community, indicated by the concentration of the patients on the map (Figure 2), illustrated high risk for local outbreaks and would then trigger home visits by social workers automatically.

### Clinical Decision Support and Prioritization of Resources

A clinical decision support system based on an inhospital mortality prediction system was built for patients with COVID-19 to improve the clinical care, decrease death risk, and prioritize limited medical resources. Based on the Multilobular Infiltration, Hypo-Lymphocytosis, Bacterial Coinfection, Smoking History, Hyper-Tension and Age (MuLBSTA) [18] scoring system, which is a partially validated prediction system for the inhospital mortality of patients with COVID-19, 36 patients out of the 383 cases were classified as high-risk (MuLBSTA score ≥12). They were either relocated to the single hospital in the area that had an intensive care unit or screened with important biochemical markers more frequently [18,19]. To the best of the authors’ knowledge, this is the first practical use of a mortality risk prediction system specifically for patients with COVID-19.

### Follow-Up for Discharged Patients

We used the social media platform to register the discharged patients and required the patients to report their symptoms daily in the 2 months after discharge. After the follow-up system was initiated, 100% coverage was achieved within 3 days. The reported recurrence of symptoms such as high fever was linked with home visits by social workers inside communities and readmission to hospitals.

## Discussion

The system had strengthened the checkpoints across the entire chain of COVID-19 control, including early discovery through symptom surveillance covering almost the whole population, early report by active information channel directly connected with follow-ups, early isolation strengthened by spatial tracking, and early treatment enabled by the clinical decision support system.

### The Effects of HHS for COVID-19

One powerful function of the hybrid system was the enhancement of the full spectrum for the management of COVID-19. The first step was the deployment of the syndromic surveillance platform, covering a large population through their mobile devices, to direct and empower the implementation of public health countermeasures. Geospatial data were also used to analyze the potential close contact history and monitor the local small-scale outbreaks. The second step was to use the inhospital mortality prediction system to direct the clinical interventions of patients and to allocate the limited resources. The third step was the seamless coverage of patients at different stages, including high-risk of infection, isolation under medical observation, admitted, critically ill, discharged, or follow-up cases. All of these management actions helped decision making for controlling the disease effectively in Honghu, which was under pressure due to the large numbers of people who traveled back from Wuhan (over 50,000), and the severe lack of medical staff and public health experts at the onset of the outbreak.

### Facilitating Factors for the Adoption of HHS

The most important factor contributing to the successful building and adoption of HHS was the deep involvement of multiple stakeholders. Experts, including those from public health, clinical medicine, medical informatics, data analytics, decision science, and other backgrounds, provided methodology guidance, clinical expertise, and research support. This helped to convince the residents who had high faith in the professionals from the national-level medical institutes such as Nanfang Hospital of Southern Medical University. Strong coordination by the local government, including the mobilization of social workers, public media exposure of the HHS gateway, and connecting services including transportation and social services, increased the coverage to over 95%, which has been traditionally difficult for a city with a population close to 1 million. Active participation and engagement by the residents were also crucial. The willingness to share their health status data during the COVID-19 epidemics was the basis for the accomplishment of the platform. Last but not least, the technology companies allocated enough resources to the local sites and provided mature technologies and protocols for implementation. During the 72 hours of building the system, a team of over 40 experts were involved, and they took shifts to make sure that progress was made every hour. It required delicate coordination and project management, dedication from each team member, and strong support on the organizational level to make sure that the contribution was appropriately recognized.

### Relevant Issues for the Migration

Internet infrastructure and technology availability are the key issues. The high penetration rate of WeChat in China provided the foundation for successful implementation of HHS [20]. Similar social media platforms such as Line and Facebook may also be suitable solutions in different countries or areas, based on an evaluation of the penetration and the active use. Cost-effectiveness analysis is also important for decision making when building new systems. Using existing open platforms also helped control the costs, which was about 3 million CNY (US ~$431,000) for HHS, including cloud-based virtual resource, labor, and other costs. The effectiveness can be more significant in areas where the infection rate is high and social distancing and traffic control are making traditional syndromic surveillance impossible [21]. Resources for implementation of the new system, especially when connecting with hospital information systems, and further data analytics would also be challenging and require coordination and collaboration between local and external resources.

### Limitations of the Study

This study has several limitations. The lack of internal or external validation of the system was caused by the emergent situation during the epidemics, the ethics dilemma, and limited resources. The sample size was not large enough to draw statistical inferences because the scope of the system was within the city of Honghu. The proposed HHS strategy can be adapted by other researchers, innovators, or authorities to develop localized or national solutions to respond to the urgent situation and help to prioritize health care resource allocation, but the clinical effectiveness of the statistical methods used in this system still requires additional studies in the future. The HHS has several deficiencies. The national ID of each participant was inputted by users manually and mistakes may exist. This can be corrected by collecting the photocopies of the ID card and automatically extracting the ID number. Moreover, because of the limited time, the system was not friendly enough for users, as mentioned in the feedback review from some government staff, and did not support access from mobile devices. Further development can help improve these existing limitations.

### Conclusion

Based on the field study in Honghu city, the HHS has been observed to be effective and feasible for COVID-19 surveillance and control. It helped strengthen the checkpoints on the full chain of COVID-19 control, including “early test, early report, early isolation, and early treatment” during the outbreak and the long-term follow-up after the epidemics. As we are still in the early stage of applying informatics systems for tackling the emerging COVID-19 pandemic, it is worth mentioning that the statistical methods used in this study require further analysis to confirm its clinical effectiveness and appropriateness. The integrated informatics technologies, cost-effective solutions, and fast deployment provided the base for its replication in areas where COVID-19 is outbreaking and for similar disease pandemics in the future.

## Appendix

Multimedia Appendix 1 Honghu project information technology infrastructure configuration.

Multimedia Appendix 2 Informed consent form used in this study in the Chinese language, as well as the corresponding English translation based on Google Translate for non-Chinese readers as a reference.

## Abbreviations

COVID-19: coronavirus disease
HHS: Honghu Hybrid System
ICD: International Classification of Diseases
MuLBSTA: Multilobular Infiltration, Hypo-Lymphocytosis, Bacterial Coinfection, Smoking History, Hyper-Tension and Age

## References

[1] Munster VJ, Koopmans M, van Doremalen N, van Riel D, de Wit E (2020). A novel coronavirus emerging in China - key questions for impact assessment. N Engl J Med.

[2] Simonsen L, Gog JR, Olson D, Viboud C (2016). Infectious disease surveillance in the big data era: towards faster and locally relevant systems. J Infect Dis.

[3] Houlihan CF, Whitworth JA (2019). Outbreak science: recent progress in the detection and response to outbreaks of infectious diseases. Clin Med (Lond).

[4] Abidi SSR, Abidi SR (2019). Intelligent health data analytics: a convergence of artificial intelligence and big data. Healthc Manage Forum.

[5] Dong X, Boulton ML, Carlson B, Montgomery JP, Wells EV (2017). Syndromic surveillance for influenza in Tianjin, China: 2013-14. J Public Health (Oxf).

[6] Bansal S, Chowell G, Simonsen L, Vespignani A, Viboud C (2016). Big data for infectious disease surveillance and modeling. J Infect Dis.

[7] Salathé M (2016). Digital pharmacovigilance and disease surveillance: combining traditional and big-data systems for better public health. J Infect Dis.

[8] Nelli L, Ferguson HM, Matthiopoulos J (2019). Achieving explanatory depth and spatial breadth in infectious disease modelling: integrating active and passive case surveillance. Stat Methods Med Res.

[9] Huang C, Wang Y, Li X, Ren L, Zhao J, Hu Y, Zhang L, Fan G, Xu J, Gu X, Cheng Z, Yu T, Xia J, Wei Y, Wu W, Xie X, Yin W, Li H, Liu M, Xiao Y, Gao H, Guo L, Xie J, Wang G, Jiang R, Gao Z, Jin Q, Wang J, Cao B (2020). Clinical features of patients infected with 2019 novel coronavirus in Wuhan, China. Lancet.

[10] Lu R, Zhao X, Li J, Niu P, Yang B, Wu H, Wang W, Song H, Huang B, Zhu N, Bi Y, Ma X, Zhan F, Wang L, Hu T, Zhou H, Hu Z, Zhou W, Zhao L, Chen J, Meng Y, Wang J, Lin Y, Yuan J, Xie Z, Ma J, Liu WJ, Wang D, Xu W, Holmes EC, Gao GF, Wu G, Chen W, Shi W, Tan W (2020). Genomic characterisation and epidemiology of 2019 novel coronavirus: implications for virus origins and receptor binding. Lancet.

[11] Li Q, Guan X, Wu P, Wang X, Zhou L, Tong Y, Ren R, Leung KS, Lau EH, Wong JY, Xing X, Xiang N, Wu Y, Li C, Chen Q, Li D, Liu T, Zhao J, Liu M, Tu W, Chen C, Jin L, Yang R, Wang Q, Zhou S, Wang R, Liu H, Luo Y, Liu Y, Shao G, Li H, Tao Z, Yang Y, Deng Z, Liu B, Ma Z, Zhang Y, Shi G, Lam TT, Wu JT, Gao GF, Cowling BJ, Yang B, Leung GM, Feng Z (2020). Early transmission dynamics in Wuhan, China, of novel coronavirus-infected pneumonia. N Engl J Med.

[12] Zhu N, Zhang D, Wang W, Li X, Yang B, Song J, Zhao X, Huang B, Shi W, Lu R, Niu P, Zhan F, Ma X, Wang D, Xu W, Wu G, Gao GF, Tan W, China Novel Coronavirus Investigating Research Team (2020). A novel coronavirus from patients with pneumonia in China, 2019. N Engl J Med.

[13] Payne PRO, Lussier Y, Foraker RE, Embi PJ (2016). Rethinking the role and impact of health information technology: informatics as an interventional discipline. BMC Med Inform Decis Mak.

[14] SNOMED.

[15] (2020). LOINC.

[16] (2020). Centers for Disease Control and Prevention.

[17] Gong M, Wang S, Wang L, Liu C, Wang J, Guo Q, Zheng H, Xie K, Wang C, Hui Z (2020). Evaluation of privacy risks of patients' data in China: case study. JMIR Med Inform.

[18] Guo L, Wei D, Zhang X, Wu Y, Li Q, Zhou M, Qu J (2019). Clinical features predicting mortality risk in patients with viral pneumonia: the MuLBSTA score. Front Microbiol.

[19] Chen N, Zhou M, Dong X, Qu J, Gong F, Han Y, Qiu Y, Wang J, Liu Y, Wei Y, Xia J, Yu T, Zhang X, Zhang L (2020). Epidemiological and clinical characteristics of 99 cases of 2019 novel coronavirus pneumonia in Wuhan, China: a descriptive study. Lancet.

[20] CINIC (2019). China Internet Network Information Center.

[21] Feldstein LR, Ellis EM, Rowhani-Rahbar A, Hennessey MJ, Staples JE, Halloran ME, Weaver MR (2019). Estimating the cost of illness and burden of disease associated with the 2014-2015 chikungunya outbreak in the U.S. Virgin Islands. PLoS Negl Trop Dis.

