# Supplementary material for: Cloud-Based System for Effective Surveillance and Control of COVID-19: Useful Experiences From Hubei, China
Source: J Med Internet Res. 2020 Apr 22;22(4):e18948. doi: 10.2196/18948 (PMC7179239; doi:10.2196/18948)
Supplement: Multimedia Appendix 1 [file jmir_v22i4e18948_app1.doc]

**Multimedia Appendix 1: Honghu Project IT Infrastructure Configuration**

| **System** | **Item** | **Description** | **OS** | **Qty.** |
| --- | --- | --- | --- | --- |
| **Vinci System** | 1. Database Server | CPU: 16-Core, Memory: 96GB, Storage: 100GB (System) 4TB (Data) | CentOS 7.4 | 2 |
|  | 1. Application Sever | CPU: 16-Core, Memory: 64GB, Storage: 100GB(System) 1TB(Data), Bandwidth: 20Mbps (billing on actual traffic) | Windows Server 2012 | 6 |
|  | 1. Front Server | CPU: 8-Core, Memory: 16GB, Storage: 100GB(System) 1TB(Data), Bandwidth: 20Mbps (billing on actual traffic) | Windows Server 2012 | 1 |
| **Resident Health Data Platform** | 1. Keepalived   +  Nginx | CPU: 4-Core, Memory: 8GB, Storage: 100GB(System) 100GB(Data), Bandwidth: 500Mbps (billing on actual traffic) | Linux | 2 |
|  | 1. Tomcat | CPU: 4-Core, Memory: 8GB, Storage: 100GB (System) 100GB(Data), Bandwidth: 100Mbps (billing on actual traffic) | Windows Server 2012 | 15 |
|  | 1. Database Server | CPU: 8-Core,  Memory: 16GB,  Storage: 100GB(System) 1TB(Data) | Linux | 2 |
| **Patient Follow Up Platform** | 1. Database Server | CPU: 16-Core, Memory: 64GB, Storage: 100GB(System) 6TB(Data) | Linux | 2 |
|  | 1. Application Sever #1 | CPU: 16-Core, Memory: 32GB, Storage: 100GB(System) 1TB(Data) | Linux | 2 |
|  | 1. Web Server | CPU: 16-Core, Memory: 32GB, Storage: 100GB(System) 1TB(Data), Bandwidth:100Mbps (billing on actual traffic) | Linux | 1 |
|  | 1. Application Sever #2 | CPU: 16-Core, Memory: 16GB, Storage: 100GB(System) 300GB(Data), Bandwidth: 4Mbps (billing on actual traffic) | Windows | 1 |
|  | 1. Jump Server | CPU: 1-Core, Memory: 4GB, Storage: 40GB(System) 200GB(Data), Bandwidth: 4Mbps (billing on actual traffic) |  | 1 |
|  | 1. Next Generation Firewall | Advanced Version, Anti-Virus, Single-Mode |  | 1 |
