# Supplementary material for: Cloud-Based System for Effective Surveillance and Control of COVID-19: Useful Experiences From Hubei, China
Source: J Med Internet Res. 2020 Apr 22;22(4):e18948. doi: 10.2196/18948 (PMC7179239; doi:10.2196/18948)
Supplement: Multimedia Appendix 2 [file jmir_v22i4e18948_app2.docx]

**Multimedia Appendix 2: Informed consent form used in this study in the Chinese language, as well as the corresponding English translation based on Google Translate for non-Chinese readers as a reference.**

**收集采集诊疗数据的知情同意书**

**尊敬的患者：**

请您仔细阅读本知情同意书并慎重做出是否同意您本人医疗数据用于未来研究的决定。参加这部分研究完全是您自主的选择，当研究人员和您本人讨论知情同意书的时候，您可以让他/她给您解释您看不明白的地方。我们鼓励您在做出参与此项研究的决定之前，和您的家人及朋友进行充分讨论。您可以自主决定您的信息是否能够保存，保存年限，是否需要匿名化保存，以及将来可以用于哪些研究。

一、**研究背景和研究目的：**为了提高新型冠状病毒肺炎的治愈率，降低病死率，防止出现轻型转重型的情况，本项目拟采集住院患者各项临床数据，深入研究新型冠状病毒肺炎患者临床特征、病情加重因素及致死因素，并建立起由大数据、人工智能为技术核心的重大疫情评估和预警系统，以便指挥部及时掌握各医院的情况，评估轻重，分类人群，科学救治。同时也为疫情结束后制定重大急性传染病防控策略，开发新的风险评估和预警模型提供重要依据。

**二、您需要做什么：**如果您愿意参加本研究，我们将采集您各项临床数据，包括医疗记录和健康记录。

**三、数据采集用途：**您的数据将来会用于与新型冠状病毒肺炎相关的科学研究。

四、**研究结果反馈：**数据采集仅仅是出于科学研究目的。如果在将来的研究中确实发现对您健康有意义的结果，研究者可能会联系您告知您相关的发现，但这不是必需的。

**五、预期受益和花费：（1）**您不会有直接获益，但使用您的可识别信息和数据进行研究可能会帮助我们理解健康和疾病、促进医疗水平的提高、促进诊疗方法更加安全或有效，并拓展新的科学知识。**（2）**参加研究您不需要支付任何与研究相关的花费。您的可识别信息可能会被用于提供促健康服务。如果出现这些情况，我们不会告知您或给您支付任何费用或补偿，但大部分数据的使用不会给任何人带来商业产品或利益。

**六、预期风险：**数据的采集不会给您带来任何额外不适。您的相关信息可能会有隐私泄露的风险，但我们会尽最大努力保护您的信息避免未被授权的其他人接触，包括去除那些很容易识别您的信息，您的可识别信息或可识别的数据被未授权的人看到的风险是极低的。

**七、数据的保存：**您的数据和信息将存储于洪湖市新冠肺炎防控综合信息平台。用于研究的相关信息的保存将是长期的，目前还不能准确推测保存时间。如果由于各种原因需对数据进行销毁，我们进行统一销毁。

**八、相关数据的使用：**您的相关数据将提供给本院研究人员或因为科研需要提供给合作单位使用。

**九、隐私保护：**系统使用权限有严格的要求，我们会在法律规定范围内保护您的个人隐私，涉及诊疗的信息会及时的与医务人员沟通，涉及科研工作，如将研究成果发表等，我们将严格按照匿名化处理，您的个人可识别信息不会出现在发表刊物上，当出现新的技术时会及时更新安全保护措施。如果数据需转移给第三方进行研究使用，我们会对第三方的资质进行审核，确保数据存放的安全性。为确保研究依照规定进行，必要时政府管理部门或伦理委员会的成员按规定可以在研究单位查阅您的个人资料。

**十、数据的转移：**数据不能转移用于科研以外的事务性工作。如转移给合作单位或第三方机构，我们会对合作单位或第三方的资质进行审核，并在合作协议中规定相关数据仅可用于科学研究，不得擅自用于其他用途，且数据的使用须严格按照知情同意、研究方案和相关法律法规的规定。

**十一、是否随时可终止储存及相关联系方式**

即使您做出同意的决定，也是可以随时改变您的决定的，但是有一定的限制。如果您改变决定或有与本研究相关的任何问题，请通过电话联系刘医生020-61687661。您的可识别信息将不能再被用于研究。但是，在您改变决定前已经开始的研究，您的信息仍将继续使用。如果事先已征得您的同意并经过伦理委员会审批，您的可识别信息已转移给第三方研究者，研究机构或公司，可能不能限制他们继续使用或是开展新的研究。

**知情同意声明**

1. 我已经阅读并理解了本知情同意书的全部内容。
2. 我有机会提问而且所有问题均已得到解答。
3. 我理解参加本活动完全是自愿的。我也明白，我也有退出的权利，我的任何医疗待遇与权益不会因此而受到影响。
4. 我知道签名并不意味可以免去任何费用、应尽责的事项。
5. 我将得到这份知情同意书的副本，上面包含我和研究者的签名。

**我同意我的数据可以捐赠给南方医科大学南方医院并同意所捐献数据用于医学研究，为早日攻克疾病和病患医治作贡献。**

**患者**签名： 日期：

联系电话：

法定代理人签字： 日期：

联系电话：

（注：如果患者无行为能力或限制行为能力时，如纳入精神障碍/意识不清等弱势群体则需法定代理人在以下法定代理人签字处签名）

公平见证人签名： 日期：

联系电话：

（注：仅当可能纳入有知情能力，但无法阅读文本的患者（如：文盲、视力障碍）时，才需要公平见证人签名，见证人知情时研究者最好留取视频材料作为知情证据）。

**研究者声明**

我已准确地将这份文件告知患者，他/她准确地阅读了这份知情同意书，并证明该患者有机会提出问题，他/她是自愿同意的。

研究者签名： 日期：

联系电话：

**Informed consent for data collection**

**Dear patient:**

Please read this informed consent carefully before making a decision to agree to share your medical data for future research. Participating in this part of the study is entirely your own choice. When the researcher discusses the informed consent with you, you can ask him/her to explain to you what you do not understand. We encourage you to fully discuss with your family and friends before making a decision to participate in this research. You can independently decide whether your information can be saved, how long it will be kept, whether it needs to be kept anonymous, and what research can be used in the future.

1. **Research background and research purpose :** In order to improve the cure rate of the COVID19, to reduce the case fatality rate, and to prevent the occurrence of mild to severe disease, this project intends to (1) collect various clinical data of inpatients, (2) to deeply study the clinical characteristics of patients with new coronavirus pneumonia, factors that aggravate the disease and lethal factors, and (3) to establish a major epidemic assessment and early warning system with big data and artificial intelligence as the core technology, so that the command department can timely oversee the situation of each hospital, assess the severity, classify the population, and provide reasonable treatment. At the same time, it also provides an important basis for the development of major acute infectious disease prevention and control strategies after the end of the epidemic, and the development of new risk assessment and early warning models.

**2. What you need to do:** If you are willing to participate in this study, we will collect all your clinical data, including medical records and health records.

**3. Purpose of data collection:** Your data will be used for scientific research related to COVID19 in the future.

4, **Results feedback:** Data collection is solely for scientific research purposes. As if we did find a healthy meaningful result in future studies, researchers may contact you to let you know of the discovery, but this is not essential.

**5. Expected Benefits and Expenses: (1)** You will not have direct benefits but using your identifiable information and data to conduct research may help us understand health and disease, promote the improvement of medical standards, and promote safer diagnostic methods effective, and expand new scientific knowledge. **(2)** You do not need to pay any research-related expenses to participate in the research. You can identify other information may be used to provide pro-health services. If these situations occur, we will not inform you or pay you any fees or compensation, but most of the use of data will not bring commercial products or benefits to anyone.

**6. Expected risks:** The data collection will not bring you any additional discomfort. Your relevant information may be at risk of privacy disclosure, but we will do our best to protect your information from unauthorized contact, including removing information that is easy to identify you, your identifiable information or identifiable information The risk of data being seen by unauthorized persons is extremely low.

**7. Data storage:** Your data and information will be stored on the comprehensive information platform for the prevention and control of COVID19 in Honghu City. The preservation of relevant information for research will be long-term, and it is not yet possible to accurately estimate the preservation time. If the data needs to be destroyed for any reason, we will destroy it accordingly.

**8, Data usage:** your relevant data will be available to researchers or hospital because of the need to provide for scientific research cooperation units to use.

**9, Privacy protection:** There are strict requirements for the use of the system. We will protect your personal privacy within the scope of the law. Information related to diagnosis and treatment will be communicated with medical staff in a timely manner, involving scientific research work, such as publishing research results, etc. It will be handled strictly in accordance with anonymization, and your personally identifiable information will not appear in published publications. When new technologies appear, security protection measures will be updated in time. If the data needs to be transferred to a third party for research and use, we will review the qualification of the third party to ensure the security of data storage. To ensure that research is conducted in accordance with regulations, members of government management departments or ethics committees can access your personal data in research units as required.

**10. Data transfer:** Data cannot be transferred for studies other than scientific research. If it is transferred to a cooperative unit or a third-party organization, we will review the qualifications of the cooperative unit or a third party, and stipulate in the cooperation agreement that the relevant data can only be used for scientific research, and may not be used for other purposes without authorization. Any data usage must strictly follow the provisions of informed consent, research programs and relevant laws and regulations.

**11. Is it possible to terminate and remove related contact information at any time**

Even though you have made your decision now, you can always change your decision with certain restrictions. If you plan to change your mind or have any questions regarding this study, please contact us Dr. Liu via telephone 020-61687661. Your identifiable information will no longer be used for research. However, your information that has been collected before the notice of change, will continue to be used. If your consent has been obtained in advance and approved by the ethics committee, your identifiable information might have been transferred to a third-party researcher, research institution or company, and it might not be able to restrict them for continuous use or for conducting new research.

**Informed consent statement**

1. I have read and understood the entire contents of this informed consent form.

2. I have the opportunity to ask questions and all questions have been answered.

3. I understand that participating in this activity is completely voluntary. I also understand that I also have the right to withdraw, and any of my medical treatment and rights will not be affected by this.

4. I know that the signature does not mean that any expenses can be waived, and due diligence may still be required.

5. I will get a copy of this informed consent form, which contains the researcher’s and my signatures.

**I agree that my data can be used by the Southern Medical University Hospital for medical research and for contributing the early treatment of patients to overcome the disease.**

Patient Signature: Date:

Contact number:

Signature of legal representative: Date:

Contact number:

(Note: If the patient is incapacitated or has limited behavioral capacity, such as the inclusion of vulnerable groups such as mental disorders / unconsciousness, the legal representative needs to sign on this form)

Fair Witness Signature: Date:

Contact number:

(Note: Only when it is possible to include patients with informed ability but unable to read the text (e.g., illiterate, visually impaired), the signature of a fair witness is required. When the witness is informed, it is best for the researcher to retain video materials as informed evidence).

**Investigator's Statement**

I have accurately informed the patient of this document. He/she has carefully read this informed consent and has the opportunity to ask questions. He/she voluntarily agreed.

Researcher's Signature: Date:

Contact number:
